# F10. DIFFERENTIAL EXPRESSION OF MICRORNAS IN CEREBROSPINAL FLUID AND PLASMA SAMPLES IN SCHIZOPHRENIA

**DOI:** 10.1093/schbul/sby017.541

**Published:** 2018-04-01

**Authors:** Juan Gallego, Eric Alsop, Todd Lencz, Kendall Van Keuren-Jensen, Anil Malhotra

**Affiliations:** 1 Weill Cornell Medical College; 2 The Translational Genomics Research Institute; 3 Zucker Hillside Hospital, Feinstein Institute for Medical Research

## Abstract

**Background:**

Genetic variants in miRNA genes and abnormalities in the concentration of microRNAs (miRNAs) in tissues and biological fluids have recently been associated with a diagnosis of schizophrenia. Most of these studies used post-mortem brain tissue or whole blood as the source of RNA. However, examination of microRNAs in cerebrospinal fluid (CSF) might provide an in vivo biomarker, more accurately reflecting expression level changes in the brain. To date, there are no studies that have investigated miRNA expression in CSF in patients with schizophrenia using small RNA-seq. In the past, our group had the opportunity of investigating the correlation between miRNA profiles in CSF and blood measured using microarray technology. Therefore, to expand our findings and use current cutting-edge technology, we measured miRNA profiles in CSF and plasma using small RNA-seq in a sample of patients with schizophrenia-spectrum disorder (SSD) diagnosis and healthy volunteers.

**Methods:**

Twenty-two SSD patients and 17 healthy volunteers underwent a lumbar puncture and a blood draw. 15–25 cc of CSF and 5–10 cc of peripheral blood were obtained from each subject. CSF and peripheral blood samples were centrifuged. CSF and plasma samples were aliquoted into 1 mL cryovials, and stored at -80C degrees. Vesicular RNA was extracted from 1 mL of CSF and plasma samples following the protocol from the Qiagen exoRNA easy kit. The BioScientific NextFlex RNA sequencing kit was used for library construction. Sequencing was done on HiSeq2500. Samples that had at least 50,000 reads going to mature miRNA sequences were included in the analysis. Differential expression analyses were conducted in R using the DESeq2 package in Bioconductor.

**Results:**

In the overall sample cohort, most subjects were male (66.7%), not Hispanic (81.0%) and black (48.7%). Mean age was 36.8 years (SD=12.3), There were no differences in age, sex, ethnicity or race between the patient and healthy control groups. In the patient group, 16 (72.7%) had schizophrenia, 5 (22.7%) had schizoaffective disorder and 1 (4.5%) had psychosis not otherwise specified. Differential expression (DE) analyses were conducted for 144 miRNAs in CSF and 354 miRNAs in plasma. After adjusting for multiple comparisons, DE analysis between patients and controls in CSF showed statistically significant higher levels in patients of miR-769-5p, miR-99b-3p, miR-107, miR-451a and miR-708-5. Similar analysis in plasma showed statistically significant higher levels in patients for miR-375, miR-204-5p, miR-942-5p, miR-6734-5p, miR-423-5p and miR-144-5p. Principal component analysis showed a clear separation between CSF and peripheral blood samples. Out of 443 miRNAs used to examine the relationship between CSF and plasma, 205 (46.3%) were detected in both plasma and CSF samples, 88 (19.9%) were detected only in CSF samples while 150 (33.9%) were detected only in plasma samples.

**Discussion:**

Five miRNAs were upregulated in CSF samples and six were upregulated in plasma samples of SSD patients compared to healthy volunteers. There was no overlap in the statistically significant upregulated miRNAs between CSF and plasma samples. Therefore, miRNA profiles in CSF and plasma have important quantitative and qualitative differences that may make them excellent, but different, candidate biofluids for biomarker discovery.

